# Composition and Expression of Genes Encoding Carbohydrate-Active Enzymes in the Straw-Degrading Mushroom *Volvariella volvacea*

**DOI:** 10.1371/journal.pone.0058780

**Published:** 2013-03-12

**Authors:** Bingzhi Chen, Fu Gui, Baogui Xie, Youjin Deng, Xianyun Sun, Mengying Lin, Yongxin Tao, Shaojie Li

**Affiliations:** 1 Mycological Research Center, College of Life Sciences, Fujian Agriculture and Forestry University, Fuzhou, China; 2 State Key Laboratory of Mycology, Institute of Microbiology, Chinese Academy of Sciences, Beijing, P.R. China; Yonsei University, Republic of Korea

## Abstract

*Volvariella volvacea* is one of a few commercial cultivated mushrooms mainly using straw as carbon source. In this study, the genome of *V. volcacea* was sequenced and assembled. A total of 285 genes encoding carbohydrate-active enzymes (CAZymes) in *V. volvacea* were identified and annotated. Among 15 fungi with sequenced genomes, *V. volvacea* ranks seventh in the number of genes encoding CAZymes. In addition, the composition of glycoside hydrolases in *V. volcacea* is dramatically different from other basidiomycetes: it is particularly rich in members of the glycoside hydrolase families GH10 (hemicellulose degradation) and GH43 (hemicellulose and pectin degradation), and the lyase families PL1, PL3 and PL4 (pectin degradation) but lacks families GH5b, GH11, GH26, GH62, GH93, GH115, GH105, GH9, GH53, GH32, GH74 and CE12. Analysis of genome-wide gene expression profiles of 3 strains using 3′-tag digital gene expression (DGE) reveals that 239 CAZyme genes were expressed even in potato destrose broth medium. Our data also showed that the formation of a heterokaryotic strain could dramatically increase the expression of a number of genes which were poorly expressed in its parental homokaryotic strains.

## Introduction

*Volvariella volvacea* (Bull.: Fr.) Singer, commonly known as the straw mushroom and the Chinese mushroom, is an important, edible, straw-degrading basidomycete fungus of the tropics and subtropics. The annual yield of the fungus was about 437,200 tons in 2008 [1]. The cultivation of *V. volvacea* uses about 4 million tons of straw per year in China, accounting for approximately 2% of the annual straw yield in China and its cultivation residues are a source of organic fertilizer with high quality for crops. Despite the economic importance of this fungus, relatively little is known about how it degrades straw and obtains nutrients, and how many enzymes take part in lignocellulose degradation. A clear grasp of the mechanism of lignocellulose degradation is important for breeding for increased straw degradation efficiency and biological efficiency, and generally to expand their immense biotechnological potential. These objectives have given impetus to the quest to sequence its genome and to characterize its gene expression. Although the number of fungal genome-sequencing projects has dramatically increased over the last few years, there is a surprising lack of information of mushroom genomes. Genome sequence information of mushrooms will help to increase the understanding of the biology, evolution, and biomedical implications of the entire fungal kingdom.

Carbohydrate-active enzymes (CAZymes), especially cellulases and hemicellulases, are involved in the hydrolysis of plant cell wall polysaccharides, and play an important role in substrate degradation processes. In recent years, the CAZymes of several biomass-degrading fungi, such as *Trichoderma reesei* [2], *Schizophyllum commune* [3], *Ganderma lucidum* [4], have been reported with their genome sequences. We recently obtained the genome sequence and transcriptome of *V. volvacea* using next-generation high-throughput sequencing technology. Prior to our study, other studies concerning cellulases,hemicellulases, β-Glucosidase and laccase of *V. volvacea* were published [5]–[10]. However, analysis of CAZymes on the basis of whole genome sequence has not been carried out on *V. volvacea*.

In this study, we annotated CAZymes encoding genes of this fungus based on *de novo* sequencing and assembly of the genome of *V. volvacea*. By comparison with 14 other filamentous fungi, we discovered some features in the composition of CAZymes in this fungus. Using the 3′-tag digital gene expression (DGE) [11], we compared the gene expression profiles among 2 homokaryotic *V. volvacea* strains PYd15 and PYd21, and one heterokaryotic strain H1521, which is a hybrid strain of PYd15 and PYd21. DGE allows millions of short RNAs and differentially expressed genes to be identified in a sample without the need for prior annotations. DGE has many advantages including greater sequencing depth, detection of unknown transcripts, practical implementation of digital tags, generation of absolute rather than relative gene expression measurements, detection of high levels of differential polyadenylation and detection of low-abundance transcripts and small changes in gene expression, that makes it particularly attractive for measuring mRNA expression and for identifying differentially expressed genes [12], [13].

## Materials and Methods

### Strains and Culture Conditions

Strains used in this study include 2 homokaryotic strains PYd21 and PYd15 and one heterokaryotic strain H1521 which was generated by a cross between PYd21 and PYd15. All *V. volvacea* strains used in this study were kindly supplied by the Mycological Research Center, Fujian Agriculture and Forestry University, Fujian, China.

*V. volvacea* strains were routinely grown at 32°C on potato dextrose agar medium (PDA) (200 g.L^−1^ of potato tissue, 20 g.L^−1^ glucose,20 g.L^−1^ agar). Liquid cultures were prepared in potato dextrose broth by shaking at 150 rpm for 7 days.

### Genome Sequencing and Assembly

DNA of strain PYd21 was used for genome-wide *de novo* sequencing. Genomic DNA was isolated using a modified CTAB (cetyltrimethylammonium bromide) approach [14] and sequenced on the Illumina Cluster Station and Illumina GAII platform at BGI-Shenzhen (Shenzhen, China). Three libraries of DNA fragments with insert sizes of 500 bp, 2000 bp and 5000 bp were generated and all of the reads produced from the three libraries were assembled using the SOAPdenovo [15] assembler (http://soap.genomics.org.cn/). Sample preparation and analytical processing (e.g., base calling) were performed by BGI-Shenzhen (http://www.genomics.cn).

### DGE Library Construction and Sequencing

Mycelia from strains H1521, PYd15 and PYd21 grown in potato dextrose broth were collected. Total RNA was extracted from mycelia using pBIOZOL Plant Total RNA Extraction Reagent, according to the manufacturer’s protocol (BioFlux). The isolated RNA was treated with RNeasy plant mini kit to remove potential genomic DNA contamination, according to the manufacturer’s protocol (QIAGEN, Germany). RNA integrity and concentration were evaluated using an Agilent 2100 Bioanalyzer (Agilent Technologies, Palo Alto, CA, USA).

RNA (6 µg) from each of the three strains was submitted to BGI-Shenzhen (Shenzhen, China) for library construction and sequencing. Sequence tag preparation was carried out using the Illumina Gene Expression Sample Prep Kit according to the manufacturer’s protocol. The mRNAs were separately isolated with oligo(dT) ligated beads, and were then reverse transcribed into double-stranded cDNA. The ds cDNAs were digested by the restriction enzymes NlaIII and MmeI. After ligation with sequencing adapters, the molecules were amplified by PCR. The three mRNA tag libraries were sequenced on the Illumina Cluster Station and Illumina HiSeq™ 2000. Image recognition and base calling were performed using Illumina Pipeline.

### Nonredundant Gene Set Preparation and Illumina DGE Tag Annotation

After filtering out tags with low quality (containing Ns) and adaptor sequences, DGE tags were mapped to the predicted genes, during which one base pair of mismatch was allowed. Ambiguous tags with multihits were excluded. The expression abundance of transcripts was measured by the number of unambiguous clean tags for each gene and then normalized to TPM (number of transcripts per million clean tags) [13], [16].

### CAZyme Annotation

The search and functional annotation for carbohydrate-active modules, including glycoside hydrolases (GH) [17], [18], [19], glycosyltransferases (GT) [20], polysaccharide lyases (PL) and carbohydrate esterases (CE) [21] in *V. volvacea* were performed by the software CAZYmes Analysis Toolkit (CAT) (http://cricket.ornl.gov/cgi-bin/cat.cgi?tab=Home) [22], using the Carbohydrate Active Enzymes (CAZy) database (http://www.cazy.org) [23].

### Detection of Differentially Expressed Genes

To identify global transcriptional changes between different samples, a previously described method [24], with some modifications by BGI-Shenzhen company [25], was utilized to identify differentially expressed genes from normalized DGE data via pairwise comparisons between differential strains (PYd15 vs H1521, PYd21 vs H1521, and PYd15 vs PYd21). Genes were defined as significantly differentially expressed if they had a P-value <0.005, a false discovery rate (FDR)≤0.001 [25], and an estimated absolute log2-fold change >2 in sequence counts across libraries. To increase the reliability, genes with Raw Intensity≤40 in both of two compared strains were filtered off.

## Results

### Formation of Heterokaryon can Rescue Growth Defects in Two Homokaryotic Strains

**Figure 1:**
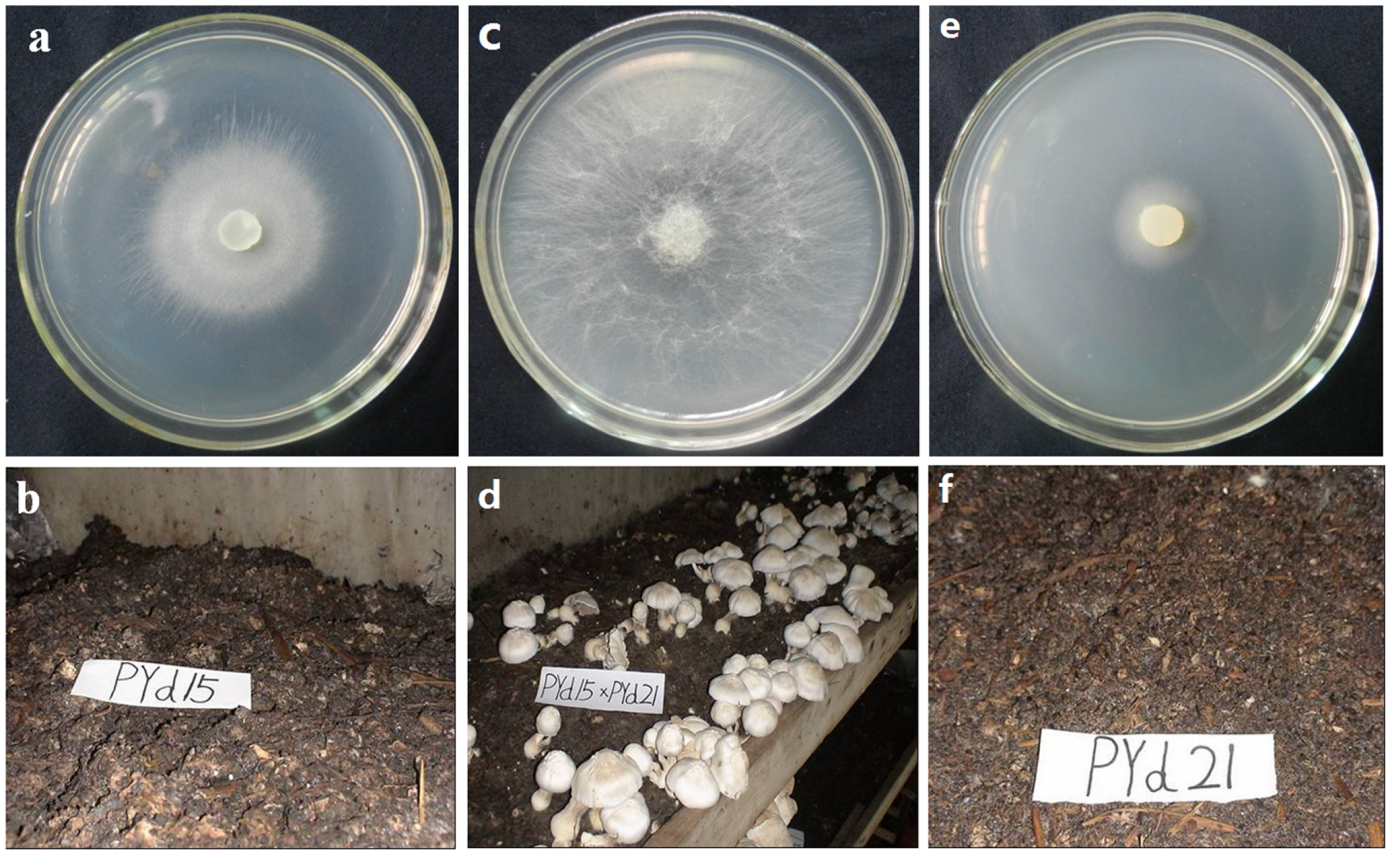
The colonial characteristics of *V. volvacea* stains PYd15(a, b), H1521(c, d) and PYd21(e, f). (a,c,e). strains were growth on PDA plates and images were captured after 4 d of growth. (b,d,f). strains were growth on a straw-based medium and images were captured after 15 d of cultivation.

Like other basidiomyces fungi, vegative mycelia of *V. volvacea* can grow in either homokaryotic or heterokaryotic form [26]. PY-1 is a widely cultivated *V. volvacea* strain in Fujian Province of China. To see the phenotypes of its homokaryotic progenies, 38 single basidiospores were isolated from PY-1. Twenty five of them displayed abnormality in colony formation (Table S1). Two single spore isolates (PYd15 and PYd21) with growth defects were chosen to cross and generated a heterokaryotic strain H1521. The growth rates of PYd15, PYd21 and the heterokaryotic strain H1521 were 0.70±0.05, 0.43±0.01 and 3.11±0.16 cm/d on PDA medium, respectively. In addition, the heterokaryotic strain H1521 had much more aerial hyphae than the two homokaryotic strains (Figure 1).

### Analysis of Genome Sequence Identified 285 CAZyme Genes in *V. volvacea*

To establish a basis for understanding molecular mechanisms that regulate *V. volvacea* growth and development, the genome of the homokaryotic strain PYd21 was sequenced by whole genome shotgun strategy and yielded 3,420 Mb clean data. A draft genome sequence of 37.2-megabase with 4.13% repeat content was assembled and deposited at DDBJ/EMBL/GenBank(http://www.ncbi.nlm.nih.gov/) under the accession no. ANCH00000000. The assembly is contained on 302 scaffolds with N50 of 499,697 base pairs (bp). A total of 11,534 ORFs were predicted using the software Eukaryotic GeneMark-ES(version2.3).

By searching the predicted amino acid sequences of *V. volvacea* genes against the Carbohydrate-Active Enzyme database (CAZy), we identified totally 285 CAZymes encoded by the genome of *V. volvacea*, including 191 glycoside hydrolases, 44 glycosyltransferases, 19 polysaccharide lyases and 31 carbohydrate esterases (the CAZymes were listed in Table S2; the predicted amino acid sequences of CAZymes are presented in Data S1).

### *V. volvacea* has a Unque CAZyme Composition

Among 15 biomass-degrading fungi with sequenced genomes (8 basidiomycetes and 7 ascomycetes), including *V. volvacea, S. commune*, *Phanerochaete chrysosporium*, *Postia placenta*, *Coprinopsis cinerea*, *Laccaria bicolor*, *Cryptococcus neoformans*, *Ustilago maydis*, *Saccharomyces cerevisiae*, *Neurospora crassa*, *Tuber melanosporum*, *Aspergillus niger*, *Piriformospora indica*, *Penicillium chrysogenum*, and *T. reesei*, *V. volvacea* ranks the seventh in the order of total CAZymes number, which is smaller than *A. niger, P. indica, P. chrysogenum, T. reesei, S. commune* and *C. cinerea* (Table 1). Compared with 7 other basidiomycetes, *V. volvacea* has a higher number of glycoside hydrolases, polysaccharide lyases and carbohydrate esterases than the average number for this lineage (161, 8, and 24, respectively). *V. volvacea* has the highest number (19) of genes encoding polysaccharide lyases (cleave uronic acid-containing polysaccharide chains) among analyzed basidiomycetes. However, *V. volvacea* has only 44 genes encoding glycosyltransferases, which is slightly below the average number (65) of this lineage.

**Table 1:** Comparison of the number of CAZymes in *V. volvacea* genome with those in other fungi genomes [3].

CAZymes	Basidiomycetes								Ascomycetes						
	V. vol.	S. com.	P. chr.	P. pla.	C. cin.	L. bic.	C. neo.	U. may.	S. cer.	A. nig.	N. cra.	T.mel.	P. ind.	P.chr.	T. ree.
GH	191	240	181	124	211	163	75	101	46	256	173	91	225	223	200
GT	44	75	66	51	71	88	64	64	68	123	76	96	73	103	103
PL	19	16	4	4	13	7	3	1	0	8	4	3	16	9	3
CE	31	30	20	13	54	20	8	19	3	25	23	14	45	22	16
Total	285	361	271	192	349	278	150	185	117	412	276	204	359	357	322

Enzymes: GH for glycoside hydrolase, GT for glycosyltransferase, PL for polysaccharide lyases, and CE for carbohydrate esterases. Species abbreviations and genome references: V. vol. for *Volvariella volvacea* (current paper), S. com. for *Schizophyllum commune* [3], P. chr. for *Phanerochaete chrysosporium* [30], P. pla. for *Postia placenta* [31], C. cin. for *Coprinopsis cinerea* [14], L. bio. for *Laccaria bicolor* [32], C. neo. for *Cryptococcus neoformans* [33]; U. may. for *Ustilago maydis* [34], S.cer. for *Saccharomyces cerevisiae* [35], N.cra. for *Neurospora crassa* [36], T. mel. for *Tuber melanosporum* [37], A.nig. for *Aspergillus niger* [38], P.ind. for *Piriformospora indica* [39], P. chr. for *Penicillium chrysogenum* [40], and T.ree. for *Trichoderma reesei* [2].

**Figure 2:**
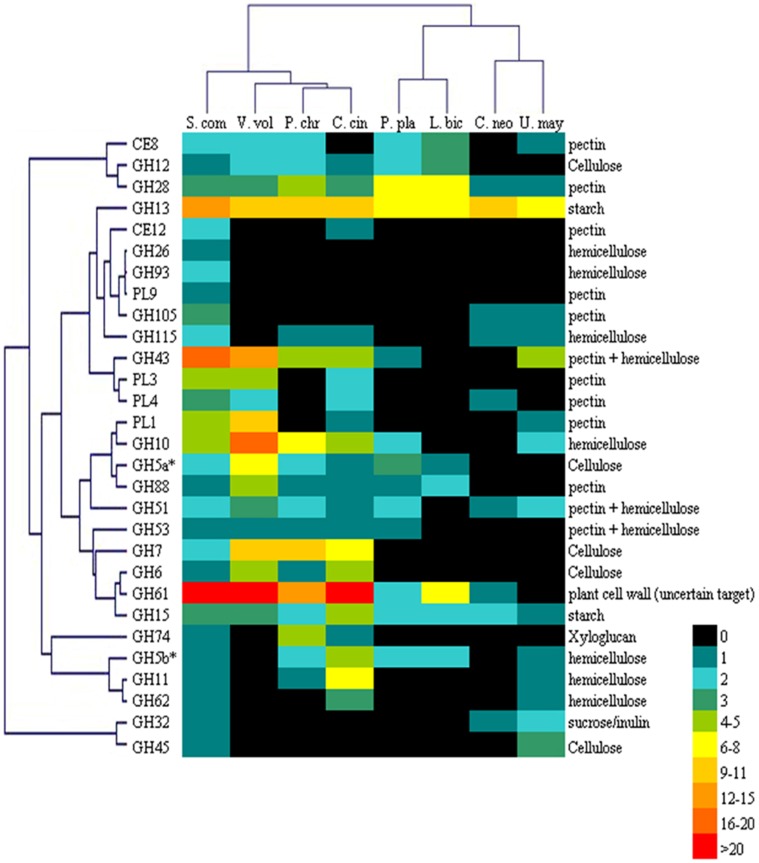
Double clustering of the carbohydrate-cleaving families of 8 basidiomycete genomes. **Top tree:** S. com. for *Schizophyllum commune*; V. vol. for *Volvariella volvacea*; P. chr. for *Phanerochaete chrysosporium*; C. cin. for *Coprinopsis cinerea*; P. pla. for *Postia placenta*; L. bio. for *Laccaria bicolor*; C. neo. for *Cryptococcus neoformans*; U. may. for *Ustilago maydis*. **Left tree:** the enzyme families are represented by their class (GH for glycoside hydrolase; PL for polysaccharide lyase; CE for carbohydrate esterase) and family number according to the carbohydrate-active enzyme database [23]. **Right side:** known substrate of CAZy families. Abundance of the different enzymes within a family is represented by a colour scale from 0 (black) to >20 (red) occurrences per species.

Among 8 basidiomycetes, white rot fungi (*V. volvacea, S. commune, P. chrysosporium, C. cinerea*) are clearly different from brown rot fungi (*P. placenta, L. bicolor, C. neoformans, U. maydis*) in both number and composition of CAZymes (Figure 2, Table S3). *V. volvacea* is rich in genes encoding enzymes that degrade pectin, hemicellulose and cellulose (Figure 2). In addition, *V. volvacea* genome is particularly rich in certain members of the glycoside hydrolase families such as GH10 (hemicellulose degradation) and GH43 (hemicellulose and pectin degradation), and the polysaccharide lyase families PL1, PL3 and PL4 (pectin degradation). However, *V. volvacea* lacks several families of glycoside hydrolase such as GH5b, GH11, GH26, GH62, GH93, GH115, GH105, GH9, GH53, GH32, GH74 and CE12, which are present in its phylogenetically related fungal species *S. commune* (Figure 2).

### Transcriptomic Profiles of Two Homokaryotic Strains with Growth Defect can be Largely Alterred by Formation of Heterokaryon

To gain an insight into mechanisms of differences in growth and development among different strains, DGE libraries of the vegetative mycelia of two homokaryotic strains PYd21 and PYd15, and their hybrid strain H1521, were constructed. The major characteristics of these libraries are summarized in Table 2. An average of 5.9 million sequence tags per library was obtained, with 182,937 distinct tag sequences. Prior to mapping these tag sequences to reference sequences, adaptor tags, low quality tags and tags of copy number = 1 were filtered out, producing an average of 5.8 million clean sequence tags per library, with 92,883 distinct clean tag sequences. By mapping DGE tags to the genome, 57,011 of 57,869 tags were found to be unambiguous tags. We filtered the clean tags mapped to multiple genes. The remaining clean tags were designated as unambiguous clean tags. The number of unambiguous clean tags for each gene was calculated as the gene expression value and then normalized to the number of transcripts per million clean tags (TPM) [13], [2]. The raw sequencing data of digital gene expression (DGE) of mycelia were submitted to Gene Expression Omnibus (GEO) database with association NO.GSE43019. (http://www.ncbi.nlm.nih.gov/geo/query/acc.cgi?token=xtoxncacgwqewnc&acc=GSE43019).

**Table 2:** Major characteristics of DGE libraries.

	PYd21		H1521			PYd15		
	Distinct Tag	Total Tag	Distinct Tag	Total Tag	Distinct Tag		Total Tag	
Raw Data	149096	5997268	199222	5838613	200492		5876147	
Tags Containing N	4485	7989	10815	34045	8115		16612	
Adaptors	185	202	125	131	442		518	
Tag CopyNum = 1	38176	38176	102656	102656	105163		105163	
Clean Tag	106250	5950901	85626	5701781	86772		5753854	
CopyNum > = 2	106250	5950901	85626	5701781	86772		5753854	
CopyNum >5	50796	5773726	38241	5566832	38525		5616866	
CopyNum >10	31336	5627851	26167	5475214	26479		5525603	
CopyNum >20	20261	5466439	18146	5358011	18269		5405543	
CopyNum >50	11261	5179615	11094	5130768	11056		5172985	
CopyNum >100	6792	4861631	7347	4862831	7341		4908306	

**Figure 3:**
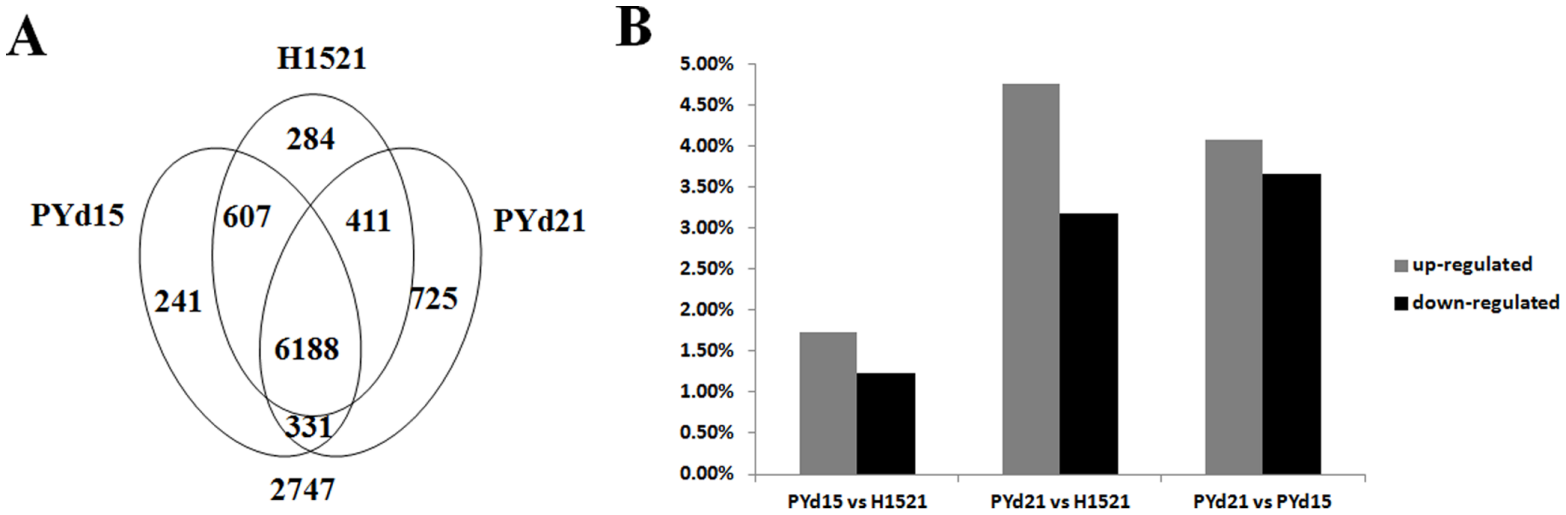
Gene expression in three strains of *V. volvacea*. (**a**) Venn diagrams showing genes expressed in the three strains. (**b**) The histogram shows the percentage of genes that are differentially expressed in the three strains of *V. volvacea*.

As shown in Figure 3, the majority of genes were either expressed in all three strains (6,188 genes) or not expressed in any of them (2747 genes). Of the 11,534 reference genes, 76.18% were expressed in at least one strain.

Transcriptomic profiles between two homokaryotic strains were dramatically different. As shown in Table S4, transcriptional levels of 470 genes in PYd15 were at least 3 times higher than in PYd21 while transcriptional levels of 422 genes in PYd15 were at least 3 times lower than in PYd21. Remarkablly, many genes with very high transcriptional levels in one homokaryotic strain had no detected transcript in another homokaryotic strain. Because *V. volvacea* is a heterothallic fungus and PYd21 and PYd15 have different homeodomain transcription factor genes at mating-type loci (unpublished data), different expression patterns in some genes might be attributed to different transcriptional regulation by mating types. In addition, since 35,389 SNP sites present between PYd15 and PYd21 genomes (unpublished data), it is not surprising that some of these SNP sites might also result in differences in gene expression.

The transcriptomic profile of the heterokaryotic strain H1521 was also significantly different from its parental homokaryotic strains. As shown in Table S5, transcriptional levels of 549 genes in H1521 were at least 3 times higher than the homokaryotic strain PYd21 while transcriptional levels of 366 genes in the heterokaryotic strain H1521 were at least 3 times lower than PYd21. Transcriptional levels of 200 genes in H1521 were at least 3 times higher than the homokaryotic strain PYd15 while transcriptional levels of 142 genes in H1521 were at least 3 times lower than PYd21 (Table S6). To be noted, transcriptional levels for 113 genes in H1521 displayed at least 3 times higher than in both homokaryotic strains. Many genes had no detectable expression in both homokaryotic strain but displayed high expression in the heterokaryotic strain (see Table 3 for the representative data), indicating the formation of heterokaryon can promote the expression of a number of genes. In contrast, 18 genes in both homokaryotic strains showed at least 3 times higher than the heterokaryotic strain H1521, suggesting the formation of heterokaryon can suppress the expression of these genes. Moreover, for 470 genes with lower transcriptional levels in PYd21 than PYd15, transcriptional levels for 266 of them in the heterokaryotic strain H1521was at least 3 times higher than those in PYd21. For 422 genes with lower transcriptional levels in PYd15 than PYd21, transcriptional levels for 53 of them in H1521 was at least 3 times higher than those in PYd15. For many genes, which had high expression in one homokaryotic strain but had no expression in another homokaryotic strain, their expression can be detected in the heterokaryotic strain (see Table 3 for the representative data). All these indicate the formation of heterokaryotic strain can alter the gene expression in a genome-wide scale, which might be the basis of the growth difference between the homokaryotic strains and the heterokaryotic strain.

**Table 3:** Effects of the formation of heterokaryon on gene expression.

Gene	Function annotation	TPM^a^-PYd21	TPM^a^-PYd15	TPM^a^-H1521	Gene	Function annotation	TPM^a^-PYd21	TPM^a^-PYd15	TPM^a^-H1521
**A. Genes with no detectable expression in PYd21**					**C. Genes with extreme low expression in both PYd21 and PYd15**				
GME11100_g	NAD(P)-binding protein	0.01	775.83	48.93	GME3377_g	predicted protein	0.5	0.01	53.49
GME7340_g	predicted protein	0.01	774.44	631.03	GME7327_g	predicted protein	0.67	2.26	144.17
GME9879_g	ectomycorrhiza-regulated small secreted protein	0.01	747.5	1.23	GME10002_g	FAD dependent oxidoreductase	0.67	5.21	67
GME7343_g	predicted protein	0.01	313.88	61.74	GME10343_g	predicted protein	0.84	8.52	574.56
GME1292_g	predicted protein	0.01	287.98	112.42	GME2777_g	predicted protein	1.01	1.22	66.3
GME3772_g	volvatoxin A2 precursor	0.01	124.96	26.48	GME5253_g	predicted protein	1.01	3.65	77.87
GME2776_g	predicted protein	0.01	39.97	7.72	GME1902_g	predicted protein	1.01	4.34	142.94
GME8956_g	predicted protein	0.01	37.54	46.13	GME3357_g	predicted protein	1.51	1.22	100.85
GME3790_g	predicted protein	0.01	32.5	19.82	GME502_g	photo-regulated tyrosinase	1.51	10.6	164.51
**B. Genes with no detectable expression in PYd15**					GME3962_g	predicted protein	2.18	2.43	72.08
GME11940_g	predicted protein	263.32	0.01	48.06	GME5044_g	predicted protein	2.69	8.52	59.1
GME4853_g	deuterolysin M35 metalloprotease	256.6	0.01	8.94	GME8904_g	thaumatin-like protein	2.69	15.82	85.06
GME11748_g	predicted protein	251.22	0.01	6.14	GME8105_g	P-loop containing nucleoside triphosphate hydrolaseprotein	2.86	6.26	63.84
GME4080_g	predicted protein	179.97	0.01	3.16	GME6749_g	predicted protein	3.19	6.95	60.51
GME2612_g	predicted protein	38.99	0.01	7.54	GME4417_g	predicted protein	3.53	1.39	1157.53
GME2589_g	predicted protein	38.48	0.01	2.46	GME5967_g	acetyl-CoA synthetase-like protein	3.53	2.26	62.96
GME5445_g	predicted protein	33.1	0.01	17.89	GME5307_g	predicted protein	3.86	1.39	80.5
GME1642_g	predicted protein	15.29	0.01	21.92	GME2317_g	predicted protein	4.2	9.21	230.28
GME3065_g	cytochrome P450	13.78	0.01	19.47	GME6508_g	predicted protein	9.58	5.04	51.74
**C. Genes with extreme low expression in both PYd21 and PYd15**					GME6820_g	predicted protein	10.59	19.81	97.34
GME3812_g	predicted protein	0.01	0.01	3284.76	GME7379_g	predicted protein	11.09	2.95	2659.7
GME8874_g	predicted protein	0.01	1.39	98.04	GME5714_g	predicted protein	11.26	4.69	126.1
GME2797_g	predicted protein	0.01	5.21	186.78	GME5639_g	predicted protein	11.93	5.21	133.99
GME2976_g	predicted protein	0.01	9.04	71.03	GME8928_g	predicted protein	15.12	17.9	75.42
GME10337_g	predicted protein	0.34	0.35	70.86	GME910_g	predicted protein	18.99	13.56	136.1

a TMP: Tags per million.

For CAZyme genes, 239 among 285 predicted genes were expressed in at least one strain (Table S7). Of these genes, 164 were expressed in all the three strains. As shown in Table S8, 15 CAZyme genes in PYd15 displayed higher transcription levels than in PYd21 while 17 CAZyme genes in PYd15 displayed lower transcription levels than in PYd21. In general, the transcriptomic profile of CAZyme genes in the heterokaryotic strain H1521 was similar to that of PYd15. Only 3 CAZyme genes showed the transcriptional differences over 3 times between H1521 and PYd15 (Table S9). In contrast, 17 CAZyme genes in H1521 displayed higher transcriptional levels than in PYd21 and 12 CAZyme genes in H1521 displayed lower transcriptional levels than in PYd21 (Table S10). Thus, transcriptional difference in CAZyme genes might not the cause to the growth difference between the heterokaryotic strain and the homokaryotic strains in PDA medium. As shown above, in addition to CAZyme genes, transcriptional levels of many other genes in the homokaryotic strains were different from those in the heterokaryotic strain. Transcriptional abnormalities in some genes might cause growth defect in the homokaryotic strains. Since formation of heterokaryon can rescue growth defects in two homokaryotic strains, their growth defects should have different genetic basis. When the homokaryon is formed, transcriptional abnormalities in one homokaryotic strain might be complemented by the other, making the heterokaryotic strain growth much better than the homokaryotic strains.

## Discussion

Based on the genome sequence, we generated a relatively complete list of CAZyme enzymes of the straw mushroom *V. volvacea* and discovered some features of this fungus in the composition of CAZymes. In addition, we showed that the transcriptomic profile of a heterokaryotic strain was completely different from those of its parental homokaryotic strains. Thus, this study is not only important for better understanding of biomass degradation of fungi but also provide an important database useful for genetic modification of the fungus in the future.

*V. volvacea* has strong activity in straw degradation but is weak for degrading the lignin component of lignocelluloses [27]. Genome sequencing and annotation of CAZymes demonstrate that, *V. volvacea* has 285 CAZymes, ranking the seventh in the number of CAZymes among 15 biomass-degrading fungi with sequenced genomes. In previous reports [3], [28], [29], [30], [31], *V. volvacea*, *S. commune*, *P. chrysosporium*, and *C. cinerea* were classified as white rot fungi, while *P. placenta, L. bicolor, C. neoformans, U. maydis* were known as brown rot fungi. The double clustering of the carbohydrate-cleaving families of these 8 basidiomycete genomes demonstrated that *V. volvacea* has a close relationship with *S. commune*, *P. chrysosporium,* and *C. cinerea,* which provide a molecular basis to support the previous classification in white rot fungi and brown rot fungi. Among 285 predicted CAZymes, transcripts for 239 of them were detected even in potato dextrose broth medium, which does not contain the best carbon source for expression of most of CAZymes. All these indicate that *V. volvacea* is genetically well equipped for biomass degradation and most of CAZyme genes are actively expressed. For many CAZyme genes, expression may be suppressed by dextrose but induced by polysaccarides. Therefore, if provided with straw-based carbon sources, more CAZyme genes should be expressed and many expressed CAZyme genes should have higher expression levels. In addition, the composition of glycoside hydrolases in *V. volvacea* is different from other basidiomycetes. Among 7 compared basiomycetes, *S. commune* has the closest phylogenetic relationship with *V. volvacea*. However, the compositions of glycoside hydrolases between *S. commune* and *V. volvacea* are obviously different. This might be a genetic basis of their different nutrient preferences: *S. commune* commonly grows on wood while *V. volvacea* grows on straw-based media. The composition of glycoside hydrolases might have close correlation with carbon utilization.

*V. volvacea* exists in either homokaryon or heterokaryon form and heterokaryotic strains usually grow better than homokaryotic strains but the molecular basis is unknown. Using DGE, we compared genome-wide transcriptional profiles among two homokaryotic strains PYd21 and PYd15 and their hybrid heterokaryotic strain H1521and found that a number of genes were differentially expressed between the homokaryotic strains. Some genes displayed very high expression levels in one homokaryotic strain but had no expression in another homokaryotic strain. However, when two genetic different homokaryotic strains fuse into one heterokaryotic strain, some defects in gene expression in each homokaryotic strain might be complemented by each other. In addition, our data also indicate that, for many genes showing low expression in both homokaryotic strains, the formation of heterokaryotic strain can significantly increase their expression. The strain-dependent gene expression reflects a very interesting regulatory phenomenon: when two genetically different homokaryotic strains are crossed to generate a heterokaryotic strain, the gene expression profile could be completely different from either of its parental strains. This further suggests that hybridization breeding can be a powerful strategy to generate *V. volvacea* strains with high yield.

To be noted, the DGE data is only based on the mycelia grown in PDA medium. If different carbon sources, such as the straw-based media commonly used for commercial production of this mushroom, are provided, the transcriptional profiles in 3 strains should be changed. Some important CAZyme genes for straw degradation might have more differences in their expression levels and provide better clues to the linkage between gene expression and growth phenotype. Although the linkage between CAZyme expression and growth remains unknown because the roles of these differentially expressed CAZyme genes in biomass degradation are not clear, it is the first report of genome-wide expression of CAZyme genes in the straw mushroom. Further deep investigation is required to clarify the detailed mechanisms of strain-dependent difference in the growth and gene expression in the future.

### Conclusion

Genome sequencing of *V. volvacea* identified 285 CAZyme genes, ranking the seventh in the number of CAZymes among 15 biomass-degrading fungi with sequenced genome. DGE analysis showed 239 CAZyme genes were expressed. A number of genes, which are poorly expressed in two genetic different homokaryotic strains, can reach to high expression levels in heterokaryotic strain.

## Supporting Information

Table S1 **The detail of 38 single basidiospores isolates from PY-1.**
(XLS)

Table S2 **285 CAZyme genes in** ***V. volvacea*** **.**
(XLS)

Table S3 **Raw data of the carbohydrate-cleaving families of 8 basidiomycete genomes.**
(XLS)

Table S4 **Differentially expressed genes between PYd21 and PYd15.**
(XLS)

Table S5 **Differentially expressed genes between H1521 and PYd21.**
(XLS)

Table S6 **Differentially expressed genes between in H1521 and PYd15.**
(XLS)

Table S7 **Transcriptional profiles of CAZyme genes in three different strains.**
(XLS)

Table S8 **Differentially expressed CAZyme genes between PYd21 and PYd15.**
(XLS)

Table S9 **Differentially expressed CAZyme genes between in H1521 and PYd15.**
(XLS)

Table S10 **Differentially expressed CAZyme genes between H1521 and PYd21.**
(XLS)

Data S1 **The predicted amino acid sequences of CAZymes in** ***V. volvacea*** **.**
(TXT)
