# Correction: Composition and Expression of Genes Encoding Carbohydrate-Active Enzymes in the Straw-Degrading Mushroom *Volvariella volvacea*

**DOI:** 10.1371/annotation/b95011bb-37ac-4770-80bf-8ba03b65bc86

**Published:** 2013-09-27

**Authors:** Bingzhi Chen, Fu Gui, Baogui Xie, Youjin Deng, Xianyun Sun, Mengying Lin, Yongxin Tao, Shaojie Li

The authors are grateful to Dr. Bernard Henrissat (Head of Glycogenomics, head of the Carbohydrate-active enzymes database (www.cazy.org), Aix-Marseille Université, France) for his comments on this article. Due to his comments, the authors wish to make the following corrections:

1. In the legend of Figure 2, this note should be included: "GH5a* represents cellulose-degrading glycoside hydrolases in the GH family 5 while GH5b* represents hemicellulose-degrading glycoside hydrolases in the GH family 5."

2. For Table S2, CE10 should be removed because this family no longer belongs to CAZymes according to the new version of Carbohydrate-active enzymes database (www.cazy.org).

3. The description of the annotation method of CAZymes, "The search and functional annotation for carbohydrate-active modules in V. volvacea were performed by the software CAZYmes Analysis Toolkit (CAT) (http://cricket.ornl.gov/cgi-bin/cat.cgi?tab=Home), using the Carbohydrate Active Enzymes (CAZy) database (http://www.cazy.org)" 

should be replaced with this description:

"The search and functional annotation for carbohydrate-active modules in V. volvacea were performed by the software CAZYmes Analysis Toolkit (CAT) (http://cricket.ornl.gov/cgi-bin/cat.cgi?tab=Home). In order to confirm the correct of annotation, we annotated the CAZymes of V. volvacea with two methods: 1. Pfam-based annotation (Search Name=aa, E-value=0.01, Bit Score=55, Use Rules=YES, Support in Family=40); 2. Sequence-based annotation (Search Name=aa-sequence, E-value=0.01, Bit Score=55, Complete genome = YES, Rules Support=40). Then the non-redundant CAZyme genes of V. volvacea were created by merging the two methods, removing the genes only annotated by one of the methods."

Recently (2013.5.6), we re-annotated the CAZymes of V. volvacea. We found 3 new CAZymes in GH128 family and 14 new CAZymes in GT2 family (GH128: GME1779_g, GME8682_g, GME9471_g; GT2: GME949_g, GME3058_g, GME3128_g, GME3510_g, GME6070_g, GME7032_g, GME7888_g, GME8577_g, GME9330_g, GME9524_g, GME9623_g, GME9765_g, GME10012_g, GME10515_g).

A new Table S2 is available here: 
